# Relations of social cognition with affective states: Insights from an expanded 2650-word database on warmth and competence

**DOI:** 10.3758/s13428-026-03085-y

**Published:** 2026-07-07

**Authors:** Dawid Żuk, Michał Chęciński, Adrianna Wielgopolan, Kamil K. Imbir

**Affiliations:** Faculty of Psychology, University of Warsaw, 5/7 Stawki St., 00-183 Warsaw, Poland

**Keywords:** Social cognition, Warmth, Competence, Affective norms, Word database

## Abstract

We expanded an existing database of affectively charged words by adding ratings of warmth and competence. We also examined the relationships between these two dimensions and previously established ones – positivity, negativity, automaticity, reflectiveness, arousal, and subjective significance. In this study, 948 participants evaluated 2650 words on warmth and competence using a modified version of the Self-Assessment Manikin scale. This allowed us to obtain ratings of perceived warmth and competence for each word. We also identified the type, direction, and strength of the relationships between warmth and competence and each affective dimension. The results provide a detailed mapping of these associations, and the database may serve as a valuable resource for future research. The findings, possible usage of our database, and limitations are discussed.

**Supplementary Information:**

The online version contains supplementary material available at 10.3758/s13428-026-03085-y.

## Introduction

The emotions we experience are inextricably linked to our social cognition. Words can evoke emotional states, which in turn influence the way we perceive our surroundings (Imbir 2017; Imbir & Pastwa, 2021). Even such seemingly minor influences as single words can shift our judgment of other people (Imbir & Wielgopolan, 2025). When people are perceived as warm and competent, observers are more likely to exhibit prosocial intentions toward them, such as the willingness to help (Fiske et al., 2007). Moreover, these dimensions affect neural mechanisms involved in social cognition, so warmth and competence significantly shape further social processes (Simon et al., 2020). It is therefore important to test how these affective states are related to the dimensions of social perception – warmth and competence. Crucially, it is essential not only to understand the direction of the relationship between these variables but also its nature. Are these associations linear or nonlinear? Does perceived warmth increase with arousal levels, or does it only rise up to a certain point before declining?

Until now, research has investigated how affective states that differ along dimensions such as valence, emotion origin, and activation influence the perception of ambiguous stimuli and human faces in terms of warmth and competence. These were experimental studies in which affective states were induced, and participants were asked to evaluate stimuli on warmth and competence dimensions (Imbir et al., 2022; Imbir, 2018; Imbir & Pastwa, 2021). However, the full network of associations between affective states and the dimensions of warmth and competence remains unknown. In particular, no studies have examined these relationships using two measures applied to the same type of stimulus – namely, emotional associations with words. For instance, is the perceived reflectiveness of a word associated with its perceived competence? In other words, is reflectiveness positively related to competence? Or is this relationship strong only for low and high reflectiveness but weak for moderate levels? And what about warmth?

To address these questions, the present study expands the existing list of 2650 Polish words characterized by affective features such as valence (positive vs. negative), activation (arousal vs. subjective significance) and the origin of emotion (automatic vs. reflective) (Wielgopolan & Imbir, 2022), by providing additional ratings on the two fundamental dimensions of social cognition: warmth and competence. Adding warmth and competence measurements for words also diversifies the existing set of tools for researchers. By updating the database with two additional components related to social cognition, we obtained warmth and competence ratings for exactly the same words for which affective state ratings were already available. This allowed us to map, in a very precise way and across a large number of items, the associations between affective states and warmth and competence.

## Warmth and competence

We propose to expand the dataset (Wielgopolan & Imbir, 2022) by incorporating the two fundamental dimensions of social perception: warmth and competence, which reflect the basic aspects of social cognition – the process by which people perceive one another, both individually and in groups (Fiske et al., 2007). It is immensely important for us to quickly gauge the intentions of newly met strangers, as well as their ability to act on those intentions, so we know what to expect and can decide how to behave – approach or avoid (Peeters, 2002; Aaker et al., 2012; Willemse et al., 2015). It is assumed that, evolutionarily, the pressure to develop the ability to judge others’ intentions was crucial for our survival (Fiske et al., 2007). The spectrum on which we make this judgment is called warmth and is linked to the valence of the impression someone makes on us. Qualities which are connected with the evaluation of warmth are, among others: communion, trustworthiness, friendliness, helpfulness, and sincerity (Abele et al., 2008; Conroy et al., 2025; Cuddy et al., 2011). A person evaluated high on the warmth spectrum is liked and seen as beneficial to others, facilitating goals valued by others (Peeters, 2002). Evaluation of warmth is an evaluation of how well someone will be able to work in a group (Abele et al., 2008; Cuddy et al., 2011; Fiske et al., 2007).

The dimension of competence relates to our judgment of how able and efficient the people we interact with would be at acting out their intentions (Fiske et al., 2007). Traits connected with competence include agency, efficiency, ability, intelligence, skill, creativity, efficacy (Abele et al., 2008; Conroy et al., 2025). Someone evaluated high on the spectrum of competence evokes respect and is seen as profitable for self (Peeters, 2002; Cuddy et al., 2011), being able to advance one’s own goals. These two dimensions, warmth and competence, are widely accepted as two universal dimensions of social cognition (Fiske et al., 2007; Wojciszke, 2005).

It is important to mention that the psychological literature includes a number of models of social cognition. Work directed at reconciling these models (Abele et al., 2021; Koch et al., 2021) proposed more dimensions of social evaluation than just warmth and competence (Yzerbyt et al., 2025). The latter, sometimes referred to as the Big Two of social cognition, can be split into facets: warmth into morality and friendliness, and competence into ability and assertiveness. Such division allows for a more precise description of social perception (Koch, 2024). However, the fundamental distinction between warmth and competence remains the core organizing principle of social cognition. Therefore, for the purpose of creating a broad lexical database, the present study focuses on these two primary dimensions.

Perceptions of an individual’s warmth and competence are shaped by multiple contextual and relational factors. However, the impact of emotion depends on the specific mechanism involved. Research suggests that a perceiver’s internal emotional involvement – such as empathically adopting a target’s perspective – tends to selectively enhance perceived warmth without necessarily influencing judgments of competence (Sevillano & Fiske, 2016). In contrast, the act of socially sharing emotions operates differently. Ludwig et al. (2022) demonstrated that disclosing emotions increases perceptions of both warmth and competence, an effect that is further amplified when the perceiver has something in common with the disclosed fact or emotion.

Research has examined the importance of evaluating warmth and competence in contexts of prejudice, often associated with migration. Evaluations of warmth and competence of members of an out-group depend on perceived threat from that out-group. Perceived competition between the in-group and the immigrant out-group lowers the evaluation of the out-group individual on both warmth and competence (Fiske et al., 2002; Constantin & Cuadrado, 2021). Also, perceiving the out-group as competent was found to evoke anger and an intention to act against the out-group (Mackie et al., 2000). On the other hand, out-groups already stereotyped as higher on warmth and competence are perceived as less threatening and less of a potential competition (Kunst et al., 2025).

Outside the prejudice literature, the condition of threat has been reported to induce searching for cues of warmth in order to affiliate with potential allies (Fay & Maner, 2015). Perceived competence can boost warmth judgment if the social target we are evaluating is relevant for a goal we want to attain (Carrier et al., 2019). Affiliation becomes more important when we share a common goal with a target perceived as competent. In fact, as an AI-human cooperation study showed, our social perception of an ally can be even more important than their objective performance (McKee et al., 2024).

The studies enumerated above suggest that the link between perceptions of warmth and competence and positive vs. negative affect is dynamic and depends on the context. The perception of warmth and competence of targets to which a contextual prejudice is tied (such as immigrants) might be burdened with the expectations of conflict of interest. The judgment of warmth and of possible allies is, in turn, dependent on the commonality of intentions.

Thus, when others’ warmth (towards us) becomes a factor of our success or even survival it’s indicators become more salient but the reverse has also been reported to be true. Observing cues of warmth in communication, which is directed at us (also commercial communication) is linked to relaxation (Su et al., 2025) and trust, which may result, e.g., in providing personal data (Aiello et al., 2020).

Information about links to automaticity and reflectiveness of warmth and competition judgments comes partly from empathy research, which assumes division into emotional and cognitive empathy, connected with System 1 and System 2 processing, respectively. A number of studies provide evidence that warmth is linked to emotional (but not cognitive) empathy, while competence is linked to both types of empathy (Sevillano & Fiske, 2016; Aue et al., 2021; Szuster & Jarymowicz, 2020). This seems in line with the dual-process account of emotions being elicited either by System 1 (automatic emotions) or by System 2 (through cognitive appraisal) and cognition being linked to System 2 (Jarymowicz & Imbir, 2015, 2015).

When we become the target of social evaluation, expressing certain emotions can impact this judgement. Showing anger has been demonstrated to be an effective counteraction to a negative competence evaluation, while expressing sadness might turn around a negative warmth evaluation in our favor (Celik et al., 2016). As the literature indicates, numerous open questions remain regarding the nature of the relationships between perceived warmth and competence and a range of emotional factors, including valence (positivity vs. negativity), arousal, subjective significance, and the origin of emotion (automatic vs. reflective). These associations were obtained by assessing the emotional content of stimuli in the form of words.

## Affective norms for words

Osgood et al. (1957) proposed that the emotional character of a word can be measured using semantic differential (Ploder & Eder, 2015), a scale used for eliciting the meanings of concepts by scoring the word denoting the concept on a number of bipolar dimensions. Osgood’s research concluded that three main dimensions capture the emotional meaning of a word. Based on this conclusion, Bradley & Lang (1999) developed a list of English words (named the Affective Norms for English Words - ANEW), each of which was scored by a number of English language users and thus located on the three spaces of: valence (from pleasant to unpleasant; Russell, 1980); arousal (from calm to excited); dominance (from “in control” to “being dominated”). For example, the word “anger” was judged to be low on the dimension of valence (so, placed on the “unpleasant” side of the pleasant-unpleasant spectrum), high on the arousal dimension, and in the middle of the dominance dimension. According to this judgment, presenting the word “anger” would evoke a rather unpleasant, high-energy emotion. The scoring of words making up the ANEW dataset was done using a pictorial scale named Self-Assessment Manikin (SAM) (Lang, 1980). SAM provides a graphic presentation of the dimension, excluding the need for linguistic labels for the extremes of the dimensions. For example, the valence dimension is represented by a smiling figure for the positive end and a frowning figure for the negative end.

## Warmth and competence in words

Although the authors of previous word databases (Jarymowicz & Imbir, 2015, 2015; Wielgopolan & Imbir, 2022) also used a modified version of the Self-Assessment Manikin in the instructions for rating the intensity of affective states in words, the ratings of affective states in words might still appear more natural than the ratings of social cognition dimensions in words. We argue that just as people can be evaluated in terms of the intensity of their affective states (e.g., arousal, reflectiveness, positivity) as well as in terms of social cognition (warmth and competence), individual words can also be evaluated with respect to their associations with particular affective states (Imbir, 2016a, 2016b; Imbir, 2017; Wielgopolan & Imbir, 2022) as well as with the dimensions of warmth and competence. In fact, this could also be suggested by the results of our study, which would appear to be consistent with what one might expect based on common intuitions about words. For instance, it would be unsurprising if a word such as immaturity scored very low on the competence scale, whereas kindness might be located at the opposite end of this scale and might also receive high warmth ratings. Indeed, research suggests that kindness can support traits associated with competence in the giver, such as a sense of efficacy (Curry, 2018).

Another factor suggesting that participants are able to estimate the level of warmth from words could be the results showing the relationship between arousal and warmth. Although we expect a positive relationship between the level of arousal in words and warmth ratings, we assume that this relationship will be curvilinear and take the shape of an inverted U. The results would indicate that words with extremely high arousal will not simultaneously be rated as extremely warm – just as in social evaluations we may expect that people who are extremely aroused will not be judged as very friendly and warm, perhaps due to the increased unpredictability of their behavior. This is confirmed by our data: among the 20 words from the database with the highest arousal, only one word scored above the mean for warmth (in fact, the high-arousal words were, for example, “aggressiveness,” where the mean arousal rating was 88.68 and warmth 33.35 on a scale from 1 to 100, where higher scores indicated greater intensity of the given affect). These results are therefore consistent with expectations that, just as people rated as warm tend to have moderately high arousal levels, words rated as warm also show moderately high arousal ratings.

We also wish to point out that many words in the study may not have been obviously interpretable as associated with warmth or competence (e.g., “sponge”). Such ambiguous words likely caused ratings to cluster around the midpoint of the 1–100 scale. For instance, the mean warmth rating for “sponge” was 54.34, and for competence, it was 49.52. Nevertheless, we decided to include the entire set of words from the previous database (Wielgopolan & Imbir, 2022).

Finally, the literature also contains estimates not only of the emotional intensity of text but also of the level of social cognition present within it. Text analysis tools based on machine learning go beyond emotions and allow for estimating the intensity of issues related to social cognition in entire sentences, also taking context into account. For example, SEANCE reports thousands of indices related to emotions, cognitive processes, and social cognition (Crossley, Kyle & McNamara, 2017).

## Connecting the Affective Norms for Words with the dual-process approach

ANEW was adapted into Polish (Imbir, 2015) and named Affective Norms for Polish Words (ANPW). Besides adaptation, the dataset was also expanded by adding further dimensions (Jarymowicz & Imbir, 2015, 2015) of origin (from reflective to automatic), source (from internal to external), and subjective significance (from high to low). The three dimensions added to the dataset introduce the dual-process approach (Kahneman, 2011; Strack & Deutsch, 2004; Tversky & Kahneman, 2018; Evans, 2003; Gawronski & Creighton, 2013) to the affective norms for words. In broad strokes, the dual-process theories posit that the human mind operates using two systems (Kahneman, 2011): System 1, which is automatic, fast, heuristic, and used for processing stimuli that are directly connected to biological survival of the organism; System 2, which is deliberative, slower, and used for higher cognitive tasks. Both systems can be involved in processing emotional stimuli. System 1 would process emotions, such as a fast, intense avoidance impulse when seeing a snake. System 2 could modulate the impulse by further cognitive processing, for example, recognizing a coiled piece of thick electrical cable as being misinterpreted for a snake, which might result in self-conscious emotions stemming from observing one’s own avoidance impulse (especially when the culture of the person experiencing this event values self-control). The dimension of origin encompasses the meaning of a word-stimuli as being automatic, mainly processed by System 1 (“from the heart”; Imbir, 2016b) or reflective, mainly processed by System 2 (“from the mind”). The arousal dimension relates to System 1 processing (e.g., high arousal when seeing what seems to be a snake). Its System 2 counterpart, activating System 2 emotional processes, happens due to an evaluation of subjective significance of an event (e.g., subjective significance of one’s own intense reaction to a coiled cable, mistaken for a snake). System 2 processing is flexible and dependent on internalized rules; the emotional effect of an appraisal (Kahneman, 2011) of an event (the feeling one experiences) might differ due to the differing subjective significance of the event for different people.

Jarymowicz and Imbir (2015) (2015 proposed a taxonomy of human emotions based on the dual-process approach. System 1 can be the origin of automatic affective reactions (e.g., the above mentioned terror as a reaction to a snake on the floor, forcing a behavioral reaction of jumping back), while System 2 would be able to generate reflective emotions, involving norms and expectations (e.g., the shame felt after realizing that it’s an inanimate coil of electrical cable, due the automatic reaction infringing on one’s perceived virtue of self-control and bravery). These two types of emotions are activated in two different ways (Imbir, 2016b). System 1 activation is achieved through arousal (Bradley & Lang, 1999; Osgood et al., 1957; Russell, 2003), the amount of energy associated with certain objects (e.g., something identified as a snake or a predator would evoke high arousal, triggering the energy necessary to escape or fight the threat). System 2 can be activated by the subjective significance of an object or event to the perceiver (Imbir, 2016b; Kissler et al., 2007; van Hooff et al., 2008; Imbir & Wielgopolan, 2025). If the object or event is recognized as something relevant to their goals, the System 2 process might be activated to handle the object or event.

Wielgopolan and Imbir (2022) further developed the ANPW dataset by conceptualizing the emotional dimensions not as bipolar (i.e., negative–positive continuum) but as bivariate. This means that the emotional space is treated as three-dimensional, with two separate axes for each aspect creating it. For example, the ends of the valence dimension (positivity and negativity) are treated as independent and can co-occur. Consequently, a single emotional experience can possess degrees of both positivity and negativity, thus giving rise to a description of ambiguous emotions. For the dimension of valence, this phenomenon is called ambivalence; nevertheless, in order to fully include also the other spaces (origin and activation), the term ambiguity was used, describing any space of two orthogonal dimensions (and ambivalence being one of the kinds of ambiguity)

From a theoretical perspective, the dual-process approach allows for specific hypotheses about the link between emotional dimensions and cognitive systems. It is proposed that the axes related to automatic, diffuse affective processing, specifically the positivity axis of valence, the arousal axis, and the automatic axis of origin, are aligned with System 1. Conversely, the axes associated with potential threat or complex evaluation, namely the negativity axis of valence, the subjective significance axis, and the reflective axis of origin – are theorized to be connected with System 2.

## Relations between warmth and competence and affective states

The current study was done within the theoretical framework of the Dual Mind model of emotion (Jarymowicz & Imbir, 2015, 2015; Imbir, 2016a, 2016b). The framework provides a taxonomy of emotions centered around the distinction between automatic and reflective emotions. The framework claims that the former are activated by arousal, which is an automatic process, linked to System 1. The latter is activated by subjective significance, a controlled process, connected with System 2.

Judgments of warmth have temporal primacy over judgments of competence. Studies related to the primacy-of-warmth effect show that words related to warmth are processed faster than those related to competence (Ybarra et al. 2001), a 100-ms exposition to a face resulted in a more reliable evaluation of warmth than competence (Willis & Todorov, 2006), and that the effect also holds for trait judgments from behavior (Zhang & Wang, 2018). This finding served as an inspiration for the 2018 study by Imbir, which demonstrated that automatic emotions increase social judgment of neutral stimuli on the warmth dimension, while reflective emotions increase the competence judgment of such neutral stimuli.

Further evidence comes from the 2025 study by Imbir and Wielgopolan, in which participants experienced experimentally induced affective states of subjective significance and arousal. In the first experiment, they were asked to evaluate ambiguous stimuli presented as QR codes, while in the second, they assessed human faces for warmth and competence. The results showed that the higher the level of subjective significance activated in participants, the higher they rated the ambiguous stimuli on both warmth and competence, which was partially consistent with earlier findings. However, a completely different pattern emerged for arousal: an inverted U-shaped relationship was observed, with the highest ratings following exposure to words of moderate arousal. Imbir et al. (2022) also found that higher levels of negativity and reflectiveness enhanced competence ratings for ambiguous stimuli. In a previous study (Imbir, 2017), it was shown that increased automaticity led to higher warmth ratings, while increased reflectiveness again led to higher competence ratings.

Perceptions of warmth and competence have consequences that extend much further. For instance, Simon, Styczynski & Gutsell (2020) demonstrated that perceptions of warmth and competence not only predict helping intentions but also modulate motor resonance – a neural process associated with empathic understanding of others’ actions. These findings suggest that warmth and competence serve as mediators between initial social impressions and both behavioral and neural responses. Taken together, these findings imply that emotional states may have broader consequences, shaping not only social judgments but also the ways in which we neurally process and respond to others.

Relationships between warmth and competence have not yet been examined in relation to all affective states – namely, positivity, negativity, automaticity, reflectiveness, subjective significance, and arousal – based on such an extensive and detailed set of stimuli (i.e., 2650 words assessed in terms of both warmth and competence). Furthermore, these associations have not yet been tested regarding the shape of the relationships between warmth and competence and the aforementioned affective states. What is the nature of these relationships – are they linear, curvilinear, or do they follow another pattern? Some previous studies have also conceptualized social perception as a unidimensional construct (Imbir, 2017), with competence and warmth positioned as opposite poles of a single continuum. How, then, do these relationships manifest in greater detail – particularly when warmth and competence are treated as distinct dimensions?

Beyond its theoretical contributions, this expanded dataset offers significant practical value for experimental design. Specifically, it enables researchers to control for social-cognitive dimensions when selecting linguistic stimuli rigorously. A key application is the ability to orthogonalize variables: researchers can now create lists of words that differ significantly in valence (e.g., positive vs. neutral vs. negative) yet are matched on warmth and competence ratings. Such carefully balanced word lists can be employed in priming paradigms to induce specific affective states before participants evaluate target stimuli, such as human faces. This ensures that observed effects in affective processing studies are driven by valence itself, rather than being confounded by social-cognitive associations – a level of control that represents a methodological advancement over previous research on the influence of affect on social judgments (Imbir & Pastwa, 2021; Imbir & Wielgopolan, 2025). Furthermore, the database facilitates precise stimulus selection for social cognition paradigms, enabling independent manipulation of warmth and competence without relying on complex narrative descriptions.

## Aims and hypotheses

One of the aims of this study was to expand the existing word database evaluated for emotional content (Wielgopolan & Imbir, 2023) by adding two further dimensions – competence and warmth. This extended database will enable researchers in the future to use an additional tool, allowing, for example, the control of warmth and competence values in words when selecting stimuli for subliminal induction of subjective significance or other affective states.

Another aim of the study was to verify the relationships between warmth and competence and the remaining emotional dimensions. Specifically, we hypothesized that warmth, being evolutionarily primary, would be more strongly linked to System 1 dimensions (e.g., automaticity, positivity). In contrast, we expected competence, which requires an estimation of capability and efficiency, to be more strongly linked to System 2 dimensions (e.g., reflectiveness, subjective significance). Furthermore, we aimed to go beyond linear models to test for curvilinear relationships, particularly predicting an inverted-U shape for Arousal, where extreme activation might diminish perceived social functioning. In other words, our approach made it possible to map these relationships more precisely.

## Method

### Participants

A total of 1044 volunteers participated in the study; 96 of them filled in less than half of the questionnaire and were also removed from the data. After this removal, we analyzed data from the remaining 948 participants (774 female, 157 male, 14 other, 3 did not provide their sex). The volunteers were recruited via the university research panel SONA, all Polish native speakers and psychology students, aged between 18 and 69 (*M =* 25.05; *SD* = 7.77*).*

### Design

We used a twin research method, as used in the study by Wielgopolan and Imbir (2022). In that study, however, words were assessed on the spaces of valence, origin and activation. In this study, we supplemented the assessment of the same words with the dimensions of warmth and competence. A database of word stimuli was turned into a questionnaire, using the Qualtrics platform. The questionnaire was designed in such a way that each participant was presented with a randomized list of 106 words and was asked to assess these words on scales of competence and warmth. The order of these assessments was randomized. The random list was generated separately for each participant, so that the lists did not repeat across participants. The questionnaire used 2648 words for the competence evaluation and 2649 for warmth. Words on the warmth dimension were rated on average 29.37 times (*Me* = 29.00; *SD* = 2.09; *Min* = 23; *Max* = 42), while on the competence dimension they were rated on average 28.47 times (*Me* = 28.00; *SD* = 2.38; *Min* = 18; Max = 43). Specifically, 99.2% of the words were rated at least 26 times for warmth, and 98.3% were rated at least 26 times for competence.

### Materials

The stimuli used in our study were words listed in the database available in the supplementary materials section of the 2022 article by Wielgopolan & Imbir, which was in turn based on the ANPW_R database (Imbir, 2016a, 2016b), but used only single words and excluded states and nations.

**Fig. 1:**
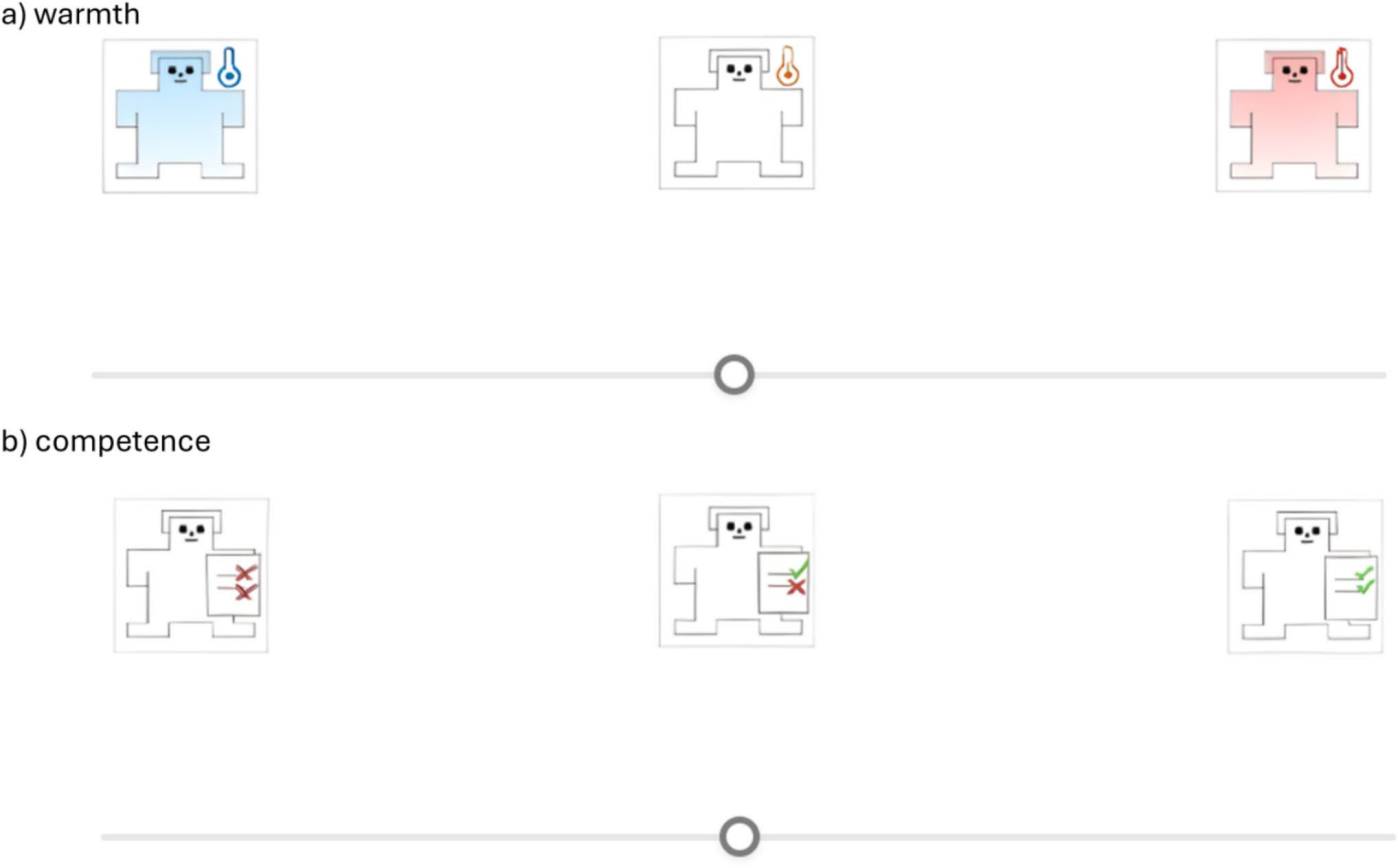
Images of the Self-Assessment Manikins used to represent varying levels of warmth and competence, as well as the sliders which could be manipulated to provide feedback about the word stimuli

The spectra of warmth and competence were described in writing, one after the other. The participants judged each word using horizontal sliders, each having a scale from 0 to 100 denoting the intensity of warmth/competence. The descriptions of the spectra also included a number of adjectives that could be used to describe the left and right edges of the sliders. The descriptions were in Polish. Besides the textual descriptions, each slider was accompanied by three pictures of simplified example emotional reactions (Self-Assessment Manikins, SAMs; Lang, 1980). The original pictures (Lang, 1980) were updated in order to reflect warmth (with a thermometer and color blue indicating low warmth and color red indicating high warmth) and competence (with the manikin holding a document with two Xs symbolizing low competence, one X and one V symbolizing medium competence, and two Vs symbolizing high competence). They are shown in Fig. 1. Descriptions of the spectrum of warmth and the spectrum of competence, translated into English:

- *Warmth relates to the intention someone expresses towards other people. The spectrum of warmth describes how intensely we feel that someone has a friendly attitude and is not threatening - i.e., is benevolent, caring, moral, and sincere. Warmth often influences our desire to become acquainted with someone.*
- *Warmth is usually used to describe people, but here we would like you to judge the extent to which words describing things, objects, states, and experiences possess (or do not possess) the quality of warmth. Please use the slider to do that.*
- *The left side of the slider could be described as cold, composed, rigid, callous, indifferent, firm, careful.*
- *The right side of the slider would be hot, sincere, helpful, impulsive, understanding, cordial, naive.*
- *Competence is how we perceive someone’s ability to act in an effective manner and reach goals. It is connected with traits such as intelligence, skills, creativity, and efficacy. Competence is judged on the basis of someone’s resources and abilities necessary to realize their plans. People seen as competent are respected and are often considered to be an authority in a given field.*
- *Competence is usually used to describe people but here we would like you to judge the extent to which words describing things, objects, stated and experiences possess (or do not possess) the quality of competence. Please use the slider to do that.*
- *The left side of the slider could be described as clumsy, in training, lost, unprepared, developing, beginner, ineffective.*
- *The right side of the slider could be described as bright, aloof, creative, intelligent, arrogant, knowledgeable, perfectionist.*

### Procedure

The participants were recruited using the SONA platform and directed to the Qualtrics questionnaire from SONA. First, a screen was presented with information about the requirement of being of legal age in order to take part in the study, the goal of the study, the SONA points given after filling in the questionnaire and the social benefits of the study, the possibility of quitting the study at any moment, the anonymity of the data collected during the study and an e-mail contact to the research team. On the next screen, the participant was asked to answer thoroughly, while being aware of their feelings. Subsequently, the participant was asked for their consent to take part in the study and to declare that they were of legal age. Demographic information was gathered next: sex (female, male, other, I do not wish to answer), age, and subject of study. The descriptions of the spectra followed. Before presenting the actual word stimuli, the participants were provided with three test sliders for each spectrum. After the screens with descriptions, the participants were asked to judge 106 words for their intensity of competence and then 106 words for their intensity of warmth. The process ended with us thanking the participant for their input and confirming that SONA points will be assigned to them. The whole questionnaire took about 35 minutes.

## Results

To answer the research questions, statistical analyses were performed using IBM SPSS Statistics 30. In the first step, we conducted an analysis of basic descriptive statistics for the intensity of warmth and competence of the examined words. Descriptive statistics were also presented for each dimension from a previous study, in which the characteristics of the same words were assessed using the same method (Wielgopolan & Imbir, 2022). In addition to warmth and competence, the ratings included spaces of valence (positivity, negativity), origin (automaticity, reflectiveness), and activation (arousal, subjective significance). In the following steps, the relationships between ratings on these dimensions and ratings of warmth and competence of the stimuli will be verified. The skewness and kurtosis values for the variables analyzed did not exceed the conventional absolute threshold of 1 (George & Mallery, 2016), indicating that the distributions deviate from symmetry only slightly. A full set of descriptive statistics for the analyzed variables is presented in Table 1.

**Table 1:** Descriptive statistics for warm, competence, and six particular emotional dimensions

Dependent variable	*M*	*Me*	*SD*	*Sk.*	*Kurt.*	*Min.*	*Max.*
Warmth	50.16	50.07	18.77	0.01	– 0.77	0.68	94.93
Competence	51.41	52.91	17.65	– 0.17	– 0.75	7.00	93.21
Positivity	37.44	34.33	21.42	0.53	– 0.54	2.30	94.37
Negativity	33.53	25.52	22.41	0.94	– 0.23	1.60	99.10
Automaticity	38.16	34.84	16.69	0.76	0.12	6.11	95.95
Reflectiveness	49.20	48.74	15.27	0.18	– 0.23	7.61	93.39
Subjective significance	41.07	37.37	18.32	0.53	– 0.59	6.00	93.21
Arousal	43.21	39.82	18.60	0.54	– 0.56	5.29	93.50

*M* – mean; *Me* – median; *SD* – standard deviation; *Sk.* – skewness; *Kurt.* – kurtosis; *Min.* – minimum value; *Maks.* – maximum value

To assess the consistency of the ratings, we calculated split-half reliability adjusted with the Spearman–Brown formula. Participants were divided into two halves based on odd and even ID numbers. We then computed the mean ratings for each word separately for both sub-samples. The correlation between the two halves was strong for both warmth, *r*(2647) =.90, *p* <.001, and competence, *r*(2646) =.87, *p* <.001. The reliability for the full sample, corrected using the Spearman–Brown formula, was *r* = 0.95 for warmth and *r* =.93 for competence, indicating excellent inter-rater agreement.

To explore the distribution of ratings across different types of words, the noun stimuli were classified into distinct semantic categories. Table 2 presents descriptive statistics for warmth and competence across these groupings. The primary division separates concrete and abstract objects. Concrete objects are further categorized into animate and inanimate entities, with animate objects subdivided into human, animal, and imaginary objects.

**Table 2:** Descriptive statistics for warmth and competence across semantic categories of nouns

Category	Dependent variable	*n*	*M*	*SD*
Concrete objects	Warmth	1578	50.94	16.38
	Competence	1578	51.05	14.73
Abstract objects	Warmth	1071	49.02	21.77
	Competence	1070	51.94	21.24
Animate objects	Warmth	394	49.84	19.34
	Competence	394	49.05	18.29
Inanimate objects	Warmth	1184	51.31	15.26
	Competence	1184	51.72	13.28
Human	Warmth	234	47.73	21.59
	Competence	234	49.90	21.27
Imaginary	Warmth	14	49.28	19.31
	Competence	14	45.84	12.96
Animal	Warmth	151	52.41	15.68
	Competence	151	47.40	13,04

*n* = number of words in a given category; *M* - mean; *SD* – standard deviation. Concrete objects are divided into animate and inanimate entities. Animate objects are further subdivided into human, animal, and imaginary referents.

Next, we conducted Pearson’s *r* correlations between the affective states of warmth and competence and the particular dimensions (Table 3). Analysis showed that warmth and competence were positively and strongly correlated, indicating that the more participants perceived words as warm, the more they also perceived them as competent (e.g., mother). Additionally, both warmth and competence were positively correlated with the positivity dimension (e.g., joy) and negatively correlated with the negativity (e.g., violent). Warmth and competence were also positively correlated with automaticity, suggesting that the more participants associated the words with automatic processing, the higher they rated them in terms of warmth and competence (e.g., passion). In contrast, reflectiveness was negatively correlated with warmth, whereas competence was positively correlated with reflectiveness. This indicates that the more participants associated the words with reflective processing, the less warmth (e.g., authority) and the more competence they attributed to them (e.g., intellect). Furthermore, both warmth and competence were positively correlated with subjective significance (e.g., love) and negatively with the arousal dimension (e.g., balance).

**Table 3:** Correlations (Pearson’s *r*) between particular dimensions

	Warmth	Competence
Competence	0.67***	-
Positivity	0.81***	0.69***
Negativity	– 0.73***	– 0.70***
Automaticity	0.49***	0.27***
Reflectiveness	– 0.21***	0.23***
Subjective significance	0.18***	0.32***
Arousal	– 0.36***	– 0.35***

Statistically significant correlations are marked with the *asterisks*; the number of asterisks corresponds to the *p* value levels of correlations: *p <.05, **p <.01, ***p <.001

To further investigate the distinct characteristics of these affective states, we compared the strength of the correlation coefficients for warmth and competence across the affective states. The analysis using Steiger’s *Z*-test revealed significant differences in the strength of associations. Specifically, warmth showed significantly stronger correlations than competence with positivity (*Z* = 12.80, *p* <.001), negativity (*Z* = 3.70, *p* <.001), and automaticity (*Z* = 15.59, *p* <.001). In contrast, competence was more strongly correlated with subjective significance than warmth was (*Z* = 9.09, *p* <.001). Furthermore, a significant difference was observed for reflectiveness (*Z* = 28.98, *p* <.001), which correlated positively with competence but negatively with warmth. No significant difference was found for arousal.

Subsequently, we examined which type of relationship best described the associations between the dimensions of warmth and competence and the spaces of valence (positivity, negativity), origin (automaticity, reflectiveness), and activation (subjective significance, arousal). We tested linear, quadratic, and cubic models, where the independent variables were either warmth or competence, and the dependent variables were the remaining dimensions. We also examined which model best described the relationship between warmth and competence.

**Fig. 2:**
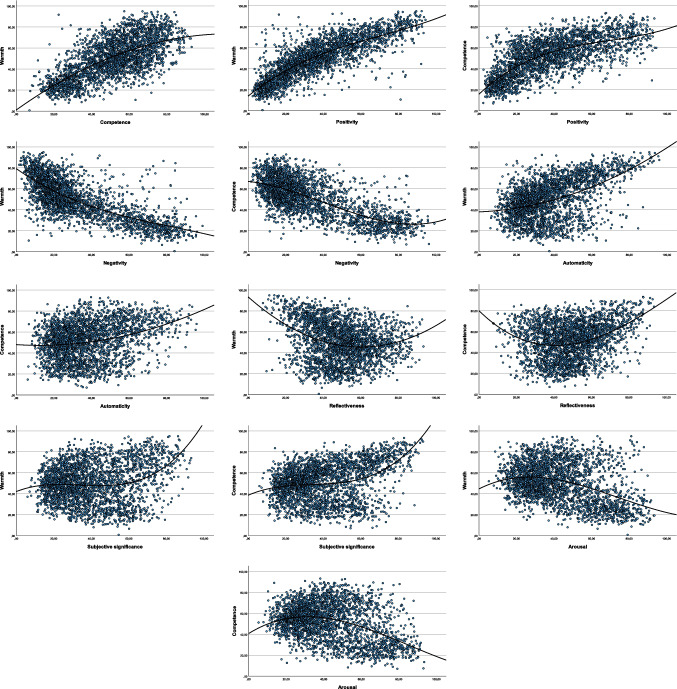
Visualization of the nonlinear relationships between warmth and competence and other dimensions; the *black line* is the estimated regression line fitted to the data

The selection of the best-fitting model was based on the highest proportion of explained variance. The results indicated that the relationship between warmth and competence was best described by a quadratic model. Similarly, the associations of both warmth and competence with arousal were also best captured by a quadratic model, as was the relationship between warmth and reflectiveness. All remaining relationships were best explained by the cubic model. Detailed coefficients can be found in Appendix  Table 4 (Table: “Regressions”), and visualizations of these models are available in Fig. 2.

In the next step (similarly to Wielgopolan & Imbir, 2022), we used the median scores of warmth and competence to create classification categories for these dimensions. We identified the following categories: words rated low on both dimensions (36.4%; *n* = 964), words high on warmth but low on competence (13.4%; *n* = 354), and words low on warmth but high on competence (13.8%; *n* = 366). We also examined the internal structure of the remaining words, characterized by high ratings on both scales. To this end, all words rated above the median on both dimensions were further divided using quartiles. In the third quartile, there were 222 words (8.4%). Whereas, 742 words (28%) fell into the fourth quartile on both dimensions.

In the following step, to examine whether there were differences in the counts of words with higher intensity of competence or higher intensity of warmth, we created an additional category. We divided the words into those with a higher intensity of warmth and those with a higher intensity of competence. It was found that 53.8% (*n* = 1424) of the words had higher competence scores, whereas 46.2% (*n* = 1224) had higher warmth scores. These counts were relatively similar. Subsequently, using chi-square tests, we assessed whether there were differences in the counts of words with higher intensity of warmth or higher competence across the different ambiguity categories. These tests were conducted separately for the group of words rated low on both dimensions (i.e., below the median–low scores category), for words in the third quartile on both dimensions (moderate scores category), and for those with at least one rating (either warmth or competence) in the fourth quartile (high scores category). For the low scores category, the chi-square test yielded a statistically significant result, χ^2^(1) = 23.97, *p* <.001. In this category, there were more words with higher competence scores (*n* = 558) than words with higher warmth scores (*n* = 406). For the moderate scores category, the chi-square test was also statistically significant, χ^2^(1) = 30.29, *p* <.001. There were more words with higher intensity of competence scores (*n* = 152) than with higher warmth scores (*n* = 70). In the final category, which included the highest scored words in terms of warmth and competence ratings, no significant differences in word counts were found, χ^2^(1) = 2.85, *p* =.091. This group contained 394 words with higher intensity of warmth scores and 348 words with higher intensity of competence scores.

Next, dependent *t* tests were conducted to compare the mean warmth and competence ratings (on continuous scales) separately for each category. In the low scores category, competence ratings (*M* = 33.86, *SD* = 10.78) were significantly higher than warmth ratings (*M* = 32.00, *SD* = 10.40), *t*(963) = 5.90, *p* <.001, *d* = 0.19.

In the moderate scores category, competence ratings were also significantly higher (*M* = 58.79, *SD* = 3.49) than warmth ratings (*M* = 56.52, *SD* = 4.30), *t*(221) = 6.31, *p* <.001, *d* = 0.42. In contrast, for the high scores category, no statistically significant difference was found between the warmth (*M* = 70.54, *SD* = 10.13) and competence ratings (*M* = 69.65, SD = 8.72), *t*(741) = 1.73, *p* =.084, *d* = 0.06.

In the next step, differences in the counts of words classified by levels of ambiguity in the valence (positivity and negativity) space were analyzed. In the low-ambiguity valence category (below the median for both positivity and negativity), there were 189 words with higher competence and 108 with higher warmth, χ^2^(1) = 22.09, *p* < 0.001. In the moderate ambiguity category, there were also significantly more competent words (*n* = 135) than warm words (*n* = 97), *χ*^2^(1) = 6.22, *p* = 0.013. For the high ambiguity category, there were no significant differences in the count of competent words (*n* = 48) versus warm words (*n* = 37), χ^2^(1) = 1.42, *p* = 0.233.

Next, analogous analyses were conducted for words categorized based on the level of ambiguity on the space of activation. The first category consisted of words with low ambiguity on both dimensions of activation (below the median for both arousal and subjective significance). In this group, words with higher competence (*n* = 443) and warmth (*n* = 469) occurred with similar counts, χ^2^(1) = 0.74, *p* = 0.389. Among words in the third quartile of results for significance and arousal (moderate ambiguity on the space of activation), the count of competent words (*n* = 425) was significantly higher than the count of warm words (*n* = 296), χ^2^(1) = 23.08, *p* < 0.001. Similarly, for high ambiguity words on the space of activation, there were no significant differences in the count of competent words (*n* = 86) versus warm words (*n* = 24), χ^2^(1) = 2.57, *p* = 0.109.

The final series of chi-square analyses tested the counts of warm and competent words across three levels of ambiguity on the space of origin (automaticity and reflectiveness). The analysis revealed that for low ambiguity on the space of origin, the count of warm words (*n* = 241) was significantly higher than that of competent words (*n* = 176), χ^2^(1) = 10.13, *p* < 0.001. At the moderate level of ambiguity, there were more competent words (*n* = 256) than warm words (*n* = 100), χ^2^(1) = 68.36, *p* < 0.001, and similarly, at the highest level of ambiguity for this space, more competent words (*n* = 86) were found than warm words (*n* = 24), χ^2^(1) = 34.95, *p* < 0.001.

## Discussion

The study aimed to examine the nature of relationships between the spaces of affective states, such as valence, activation, and origin, and the dimensions of social perception – competence and warmth. Additionally, the study aimed to expand the affectively charged word database by incorporating new dimensions – warmth and competence. Both objectives were achieved. The word database (Wielgopolan & Imbir, 2022) was updated to include these new dimensions, and the strength, direction, and shape of the relationships among warmth and competence, positivity and negativity, arousal, significance, automaticity, and reflectivity were examined.

It turned out that the obtained relationships between warmth and competence are consistent with the theory regarding the origin of affective states – as being more strongly related to either the automaticity or reflectiveness. It is assumed that warmth is more closely linked to the first, more automatic system, while competence is related to the second, more reflective system (Ybarra et al., 2001; Willis & Todorov, 2006). Analyzed affective states can also be theoretically divided into those more related to the automatic System 1 (automaticity, arousal) and those linked to the more reflective System 2 (reflectivity and subjective significance) (Jarymowicz & Imbir, 2015). The correlation results are partially consistent with this theory; however, thanks to the knowledge of the shape of these relationships, these results are consistent with the theory of the division of affective states into those more related to the first and second systems. They also allow for a more detailed understanding of the relationships between affective states and warmth and competence.

It occurred that warmth was indeed positively associated with automaticity and negatively with reflectivity. In line with that theory was also that the competence dimension showed a positive relationship with reflectivity (in the opposite direction to warmth).This may result, for example, from the fact that deeper reflection and consideration may be associated with better decisions, especially among participants with low self-reflection (Donovan et al., 2015). The negative relationship between warmth and reflectivity may, in turn, result from the notion that when we evaluate something as friendly, it does not require deeper processing. Such processing may only be necessary in threatening situations (Willis & Todorov, 2006). The positive relationship between automaticity and warmth also confirms this. If we trust someone, it is likely that we can allow ourselves more automaticity and less scanning of the environment (Bargh et al., 2012). It is worth noting that the negative relationship between warmth and reflectiveness is U-shaped. It turns out that extreme levels of reflectiveness (both high and low) are associated with high warmth scores. While possible explanations for the link between low reflectiveness and high warmth – such as trust and automaticity – have already been discussed, we hypothesize that the association between high reflectiveness and warmth may stem from the fact that when something is associated with warmth and closeness, we are more willing to allocate greater cognitive resources if necessary (Coull & Yzerbyt, 2001; Schindler et al., 2021). This reasoning is also supported by a similar pattern of results for the relationship between subjective significance and warmth.

It should, however, be noted that the relationship between warmth and automaticity is clearly stronger than the relationship between competence and automaticity. Competence is also positively related to the second affective state of origin – reflectivity. This dual association of competence with both automaticity and reflectivity indicates that competence judgments may involve both intuitive and deliberative processes, depending on the context and familiarity with the task (Imbir, 2018). The U-shaped relationship between reflectivity and competence also supports these assumptions. The positive relationship between competence and reflectivity may arise from the fact that competence is associated with certain skills, which are not always easily accessible and may sometimes require effort to acquire or learn. Thus, it seems that reflection may facilitate this process (Bargh et al., 2012). This dual-process framework is further supported by research indicating that warmth judgments are primary in social evaluations, often occurring before competence judgments. This primacy is evolutionarily advantageous, as assessing another’s intentions (warmth) is more critical for survival than assessing their abilities (competence) (Fiske et al., 2007; Ybarra et al., 2001). Interestingly, both warmth and competence were positively associated with automaticity, though the association was stronger for warmth. This suggests that while warmth evaluations are primarily automatic, competence can also be assessed automatically under certain conditions, possibly when tasks become routine or well-practiced (Bargh et al., 2012).

Another aspect worth considering is that both warmth and competence were associated in the same direction with the space of activation (arousal and subjective significance). Both warmth and competence were positively related to subjective significance. However, this relationship was moderately strong for competence and weak for warmth. Considering the fact that warmth and competence are dimensions of social perception, the stronger relationship of competence with subjective significance may arise from the idea that self-evaluations and evaluations of close others along the competence dimension have a greater impact on self-esteem than on the warmth dimension (Abele & Wojciszke, 2007). Subjective significance also needs more complex processing, which could be the reason why it is linked to competence (Hareli et al., 2013).

The relationships between warmth and competence with arousal, on the other hand, were negative and of similar strength. It is important to note that a quadratic model best describes these relationships. According to this model, both very low and very high arousal were evaluated less warmly and competently, whereas moderate arousal was rated more warmly and competently. The exact same pattern was found in the study by Wielgopolan and Imbir (2025), where an inverted U-shaped relationship was observed – with ratings being highest following words of moderate arousal. This pattern is consistent with the Yerkes–Dodson law. In the context of social perception, moderate arousal may facilitate optimal processing and evaluation of social cues, enhancing perceptions of warmth and competence (Yerkes & Dodson, 1908). This could also be explained logically, as both very high and very low arousal might have been unpleasant for participants, e.g., too boring for low arousal or too overwhelming for high arousal (Kuperman et al., 2014). A greater arousal is also associated with enhanced allocation of attentional resources (Zsidó & Kiss, 2024; Hinojosa et al., 2012) and interference effects (Aquino & Arnell, 2007).

The results obtained are also consistent with experimental studies conducted on this topic. They showed that inducing a positive affective state in the participants enhanced the evaluation of ambiguous stimuli (Japanese pictograms) on the warmth dimension, and that inducing reflectiveness in the participants enhanced the evaluation of competence to a greater extent than of warmth (Imbir & Pastwa, 2021). Participants, under the influence of increased automaticity, rated ambiguous stimuli higher in warmth than when they were in a reflective state (Imbir & Pastwa, 2021; 2017). In Imbir’s study (2018), it was found that with stronger activation of the subjective significance affective state in participants, they rated ambiguous stimuli as much more associated with competence rather than warmth. Since the ratings in that study were on a unidimensional Likert scale (1–5), where 1 indicated association with competence and 5 indicated association with warmth, these results might suggest that subjective significance was negatively related to warmth. However, in this study, which used a multidimensional approach for each affective state, we found that subjective significance is also positively related to warmth, though weaker than with competence. This discrepancy may partly be due to the measurement method, as a unidimensional scale might constrain the complexity of affective evaluations. This method of measurement alone may have significantly altered the results, especially for the affective measurements (Mauss & Robinson, 2009).

A limitation of the study is that these relationships were examined on a finite sample of 2,650 words, which, although substantial, may become somewhat outdated over time as the language used by subsequent generations continues to evolve (Croft, 2013). Another factor is the homogeneity of the sample. While the sample was large, comprising almost 1000 participants, and the variables had almost perfectly normal distributions, most participants were female psychology students. Although participants were provided with precise definitions of warmth and competence, applying these dimensions to certain words (e.g., “germ”) might have been counterintuitive. This interpretative difficulty could have led to a tendency toward midpoint ratings in cases of uncertainty, a factor that should be considered when analyzing the results for these specific items. Additionally, the use of specific anchoring adjectives (e.g., “impulsive” for warmth) might share semantic overlap with dimensions like automaticity, potentially influencing the correlation strength. However, precise definitions and visual aids were provided alongside these adjectives to minimize this influence. A significant strength of the study, however, is the systematic examination of the relationships between dimensions of social perception and affective states in the context of emotional ambiguity on a very large set of stimuli (Wielgopolan & Imbir, 2022). This provides a much more accurate picture of the relationships between these variables and also leaves for future studies a tool that other researchers can use, for example, by knowing the affective state of specific words, they can evoke these affective states while precisely controlling the intensity of other affective states (Imbir & Pastwa, 2021; Imbir, 2017; Imbir, 2018; Imbir et al., 2022).

## Future directions

In future studies, the experimental influence of specific affective states on evaluations of warmth and competence in ambiguous stimuli or faces can be tested. Using this database enables the control of warmth and competence values in words when, for example, selecting words for subliminal induction of positive affect. Valence ratings are strongly correlated with warmth, making it difficult to manipulate affect purely in experimental studies, since one may also be inducing warmth (Wielgopolan et al., 2025). The literature already provides easily accessible tools that allow researchers to select words from a word database to manipulate certain emotional dimensions while simultaneously controlling for other semantic properties of the words in an unsupervised manner – something that is very time-consuming and error-prone when done manually (Plisiecki & Sobieszek, 2024). Using such a tool would therefore make it possible to induce, for example, arousal in participants via words while controlling for the level of warmth induced.

It is also possible to examine whether there are any moderators of the impact of affective states on warmth and competence ratings, such as perfectionism or black-and-white thinking (Slaney et al., 2001). For example, it may turn out that individuals with a high level of all-or-nothing thinking might show stronger associations between their perceived arousal and their evaluations of warmth or competence. Furthermore, future research could explore how specific semantic categories (e.g., abstract vs. concrete concepts, animate vs. inanimate entities, or social roles) influence warmth and competence ratings. While the current study maintained the original database structure to ensure consistency and avoid subjective categorization, a dedicated investigation into these semantic distinctions would provide deeper insight into how different types of concepts are evaluated on these fundamental dimensions.

## Conclusion

The present study contributes to the growing body of research exploring how affective dimensions are related to fundamental aspects of social cognition. By expanding the existing dataset of words rated for warmth and competence, we provide a new tool for investigating social perceptions of verbal stimuli. Furthermore, the observed associations between affective characteristics of words – such as arousal, valence, origin, and subjective significance – and perceptions of warmth and competence support the dual-process perspective on emotion and social judgment. These findings give more possibilities for research on language, affect, and social perception by highlighting the complex interplay between emotional meaning and social evaluation.

## The data set description

All of our word stimuli are presented in Appendix Table 5 in English (Column A) and Polish (Column B). We also provide the number of letters for Polish words (Column C), mean ratings for warmth (Column D) and competence (Column E), as well as the ratings of each word on the other dimensions (Columns F, G, H, I, J, and K, respectively, for positivity, negativity, arousal, subjective significance, automaticity, and reflectiveness). To ensure full transparency and facilitate the use of the data in future research, the complete database containing the word stimuli (named Appendix Table 5) is available in the Supplementary Materials and for download in the public OSF repository at the following link: https://osf.io/t85ry/

## Electronic supplementary material

Below is the link to the electronic supplementary material.

Supplementary file1 (XLSX 251 KB)

## Data Availability

All data generated or analyzed during this study are included in this published article (Appendices  Table 4 and 5).

## Appendix

Tables 4, 5, 6, 7, 8, 9, 10, 11, 12, 13, 14, 15 and 16

**Table 4:** Quadratic regression model predicting warmth based on competence

	*B*_*1*_	*B*_*2*_
***F*** **(2;2645) = 1143.32; ** ***p*** ** < 0.001; ** ***R*** ^**2**^ ** = 0.464**		
Competence	1.28	– 0.01

Dependent variable warmth – *B*_1_ - unstandardized coefficient of linear regression; *B*_2_ - unstandardized coefficient of quadratic regression; *F* - result of variance analysis; *R*^2^. - R-squared

**Table 5:** Cubic regression model predicting warmth based on positivity

	*B*_*1*_	*B*_*2*_
***F*** **(3;2645) = 1805.10; ** ***p*** ** < 0.001; ** ***R*** ^**2**^ ** = 0.672**		
Positivity	1.45	– 0.01

Dependent variable warmth – *B*_1_ - unstandardized coefficient of linear regression; *B*_2_ - unstandardized coefficient of quadratic regression; *F* - result of variance analysis; *R*^2^. - R-squared

**Table 6:** Cubic regression model predicting competence based on positivity

	*B*_*1*_	*B*_*2*_
***F*** **(3;2644) = 965.78; ** ***p*** ** < 0.001; ** ***R*** ^**2**^ ** = 0.523**		
Positivity	1.68	– 0.02

Dependent variable competence – *B*_1_ - unstandardized coefficient of linear regression; *B*_2_ - unstandardized coefficient of quadratic regression; *F* - result of variance analysis; *R*^2^. - R-squared

**Table 7:** Cubic regression model predicting warmth based on negativity

	*B*_*1*_	*B*_*2*_
***F*** **(3;2645) = 1089.86; ** ***p*** ** < 0.001; ** ***R*** ^**2**^ ** = 0.553**		
Negativity	– 1.26	0.01

Dependent variable warmth – *B*_1_ - unstandardized coefficient of linear regression; *B*_2_ - unstandardized coefficient of quadratic regression; *F* - result of variance analysis; *R*^2^. - R-squared

**Table 8:** Cubic regression model predicting competence based on negativity

	*B*_*1*_	*B*_*2*_
***F*** **(3;2644) = 846.40; ** ***p*** ** < 0.001; ** ***R*** ^**2**^ ** = 0.490**		
Negativity	– 1.83	– 0.01

Dependent variable competence – *B*_1_ - unstandardized coefficient of linear regression; *B*_2_ - unstandardized coefficient of quadratic regression; *F* - result of variance analysis; *R*^2^. - R-squared

**Table 9:** Quadratic regression model predicting warmth based on automaticity

	*B*_*1*_	*B*_*2*_
***F*** **(2;2646) = 441.06; ** ***p*** ** < 0.001; ** ***R*** ^**2**^ ** = 0.250**		
Automaticity	0.08	0.01

Dependent variable automaticity – *B*_1_ - unstandardized coefficient of linear regression; *B*_2_ - unstandardized coefficient of quadratic regression; *F* - result of variance analysis; *R*^2^. - R-squared

**Table 10:** Quadratic regression model predicting competence based on automaticity

	*B*_*1*_	*B*_*2*_
***F*** **(2;2645) = 115.80; ** ***p*** ** < 0.001; ** ***R*** ^**2**^ ** = 0.081**		
Automaticity	– 0.11	0.00

Dependent variable competence – *B*_1_ - unstandardized coefficient of linear regression; *B*_2_ - unstandardized coefficient of quadratic regression; *F* - result of variance analysis; *R*^2^. - R-squared

**Table 11:** Quadratic regression model predicting warmth based on reflectiveness

	*B*_*1*_	*B*_*2*_
***F*** **(2;2645) = 132.20; ** ***p*** ** < 0.001; ** ***R*** ^**2**^ ** = 0.091**		
Reflectiveness	– 1.58	0.01

Dependent variable warmth – *B*_1_ - unstandardized coefficient of linear regression; *B*_2_ - unstandardized coefficient of quadratic regression; *F* - result of variance analysis; *R*^2^. - R-squared

**Table 12:** Cubic regression model predicting competence based on reflectiveness

	*B*_*1*_	*B*_*2*_
***F*** **(2;2645) = 118.822; ** ***p*** ** < 0.001; ** ***R*** ^**2**^ ** = 0.119**		
Reflectiveness	– 1.76	0.03

Dependent variable competence – *B*_1_ - unstandardized coefficient of linear regression; *B*_2_ - unstandardized coefficient of quadratic regression; *F* - result of variance analysis; *R*^2^. - R-squared

**Table 13:** Cubic regression model predicting warmth based on subjective significance

	*B*_*1*_	*B*_*2*_
***F*** **(3;2645) = 68.41; ** ***p*** ** < 0.001; ** ***R*** ^**2**^ ** = 0.072**		
Subjective significance	0.71	– 0.02

Dependent variable warmth – *B*_1_ - unstandardized coefficient of linear regression; *B*_2_ - unstandardized coefficient of quadratic regression; *F* - result of variance analysis; *R*^2^. - R-squared

**Table 14:** Cubic regression model predicting competence based on subjective significance

	*B*_*1*_	*B*_*2*_
***F*** **(3;2644) = 142.67; ** ***p*** ** < 0.001; ** ***R*** ^**2**^ ** = 0.139**		
Subjective significance	0.78	– 0.02

Dependent variable competence – *B*_1_ - unstandardized coefficient of linear regression; *B*_2_ - unstandardized coefficient of quadratic regression; *F* - result of variance analysis; *R*^2^. - R-squared

**Table 15:** Cubic regression model predicting warmth based on arousal

	*B*_*1*_	*B*_*2*_
***F*** **(3;2645) = 153.69; ** ***p*** ** < 0.001; ** ***R*** ^**2**^ ** = 0.464**		
Arousal	0.91	0.02

Dependent variable warmth – *B*_1_ - unstandardized coefficient of linear regression; *B*_2_ - unstandardized coefficient of quadratic regression; *F* - result of variance analysis; *R*^2^. - R-squared

**Table 16:** Cubic regression model predicting competence based on arousal

	*B*_*1*_	*B*_*2*_
***F*** **(3;2644) = 171.60; ** ***p*** ** < 0.001; ** ***R*** ^**2**^ ** = 0.464**		
Arousal	1.11	– 0.02

Dependent variable competence– *B*_1_ - unstandardized coefficient of linear regression; *B*_2_ - unstandardized coefficient of quadratic regression; *F* - result of variance analysis; *R*^2^. - R-squared
